# A faunistic study of genus *Chasmogenus* Sharp, 1882 of China (Coleoptera, Hydrophilidae)

**DOI:** 10.3897/zookeys.738.21711

**Published:** 2018-02-19

**Authors:** Fenglong Jia, Yu-dan Tang

**Affiliations:** 1 Institute of Entomology, Life Science School, Sun Yat-sen University, Guangzhou, 510275, Guangdong, China

**Keywords:** C*hasmogenus*, China, Hydrophilidae, new record, new species, Oriental region

## Abstract

*Chasmogenus* Sharp, 1882 is newly reported from the Chinese Mainland. A new species, *C. parorbus* **sp. n.**, is described from China (Yunnan). *Chasmogenus orbus* Watanabe, 1987 is reported from Hong Kong, the first record outside Japan. *Chasmogenus abnormalis* (Sharp, 1890) is reported from the Chinese mainland for the first time. The male genitalia of each species are illustrated. A key to the Chinese species of the genus is provided.

## Introduction

The genus *Chasmogenus* was erected by Sharp (1882) based on *Chasmogenus fragilis* Sharp, 1882. d’Orchymont (1919) treated it as a subgenus of *Helochares* Mulsant, 1844 until Fernández (1986) separated it as a valid genus. Hebauer (1992) revised all known species of the genus up to that time and recorded 24 species, with Hansen (1999) listing 26 species globally. Since then, new species have been described from the Oriental, Australian, Neotropical and Afrotropical regions (Watts 1998; Garcia 2000; Hebauer 1995, 2001, 2002, 2006; Short 2005, 2010, Short and Hebauer 2006; Clarkson and Ferreira 2014). A total of 43 species is currently known, of which 18 occur in the Afrotropical region, eleven in the Neotropic, two in the Palearctic, three in the Australian, and five in the Oriental region. *Chasmogenus abnormalis* (Sharp, 1890), known from Cambodia, Indonesia (Borneo, Java, Sulawesi, Sumatra), Sri Lanka, China (Taiwan), Thailand, Vietnam, and the Ryukyu Islands (Hebauer 1992), was considered by Hansen (1999) to occur in the Palearctic region. Based on the fauna of insects, the authors prefer to consider Ryukyu Islands as a part of the Oriental region. Therefore, *C. abnormalis* (Sharp, 1890) should be treated as an Oriental species only.

The genus has been poorly known from China up to now. Only *C. abnormalis* (Sharp, 1890) is known from Taiwan (Watanabe 1987; Hebauer 1992; Hansen 1999) and no species have been reported from mainland China to date. Since 2006, some material of *Chasmogenus* was collected by from southern China, confirming that three species occur in China. In this contribution, a new species is described and two species new for mainland of China are reported.

## Materials and methods

Specimens of each species were dissected, and the genitalia placed in a drop of glycerol on glass slides. After photography, genitalia were transferred to a plastic mount pinned with the respective specimen. Habitus photographs were taken using an Axioskop 40 compound microscope with AxioCam HRc Rev. 3/3.3v (4164×3120). Photographs of genitalia were taken using an Olympus SZX7 stereomicroscope, and subsequently combined with Auto-Montage software. The SEM photograph was taken using a Phenom Prox scanning electronic microscope. Complete label data are provided for type specimens, exact label data in English being cited for the type material (data in Chinese are translated into English). All specimens used in this study are deposited in the collection of Sun Yat-sun University, Guangzhou, China (**SYSU**).

## Taxonomy

### Chasmogenus

Taxon classificationAnimaliaColeopteraHydrophilidae

Sharp, 1882

Chasmogenus Sharp, 1882: 73. Type species: Chasmogenus fragilis Sharp.
Crephelochares Kuwert, 1890: 38. Type species: Helochares livornicus Kuwert. Syn.: d’Orchymont 1919: 148; Fernández 1986: 148.

#### Diagnosis.

The following character combinations can be used to separate *Chasmogenus* Sharp, 1882 from other genera: 1) maxillary palpi at least as long as width of head; 2) second maxillary palpomeres curved inward, apical segment almost symmetrical, as long as penultimate; 3) clypeus not concealing labrum and not expanded in front of eyes; 4) mesoventrite with rather strong median carina for entire length; 5) elytra without striae or rows of serial punctures; 6) elytra with sutural stria on posterior half; 7) posterior margin of 5^th^ abdominal ventrite with a small apical emargination.

### Chasmogenus parorbus sp. n.

Taxon classificationAnimaliaColeopteraHydrophilidae

http://zoobank.org/3496519B-82B5-42E1-B6B6-C5A4A069A99F

Figs 1–7 , 8

#### Type material.

**Holotype: CHINA. Yunnan**: male (SYSU), CHINA: Yunnan Prov., Yingjiang, Tongbiguan, Kaibangyahu, 24.58°N, 97.67°E, 1289m, 25.V.2016, Yu-dan Tang & Rui-juan Zhang leg. [transcribed from Chinese]. **Paratypes** (3 males, SYSU): 2 males, same data as holotype; 1 male., **Yunnan**, Yingjiang, Nabang, 24.75°N, 97.56°E, 239m, 27.V.2016, Yu-dan Tang & Rui-juan Zhang leg. [transcribed from Chinese].

#### Diagnosis.

**Figures 1–7.**
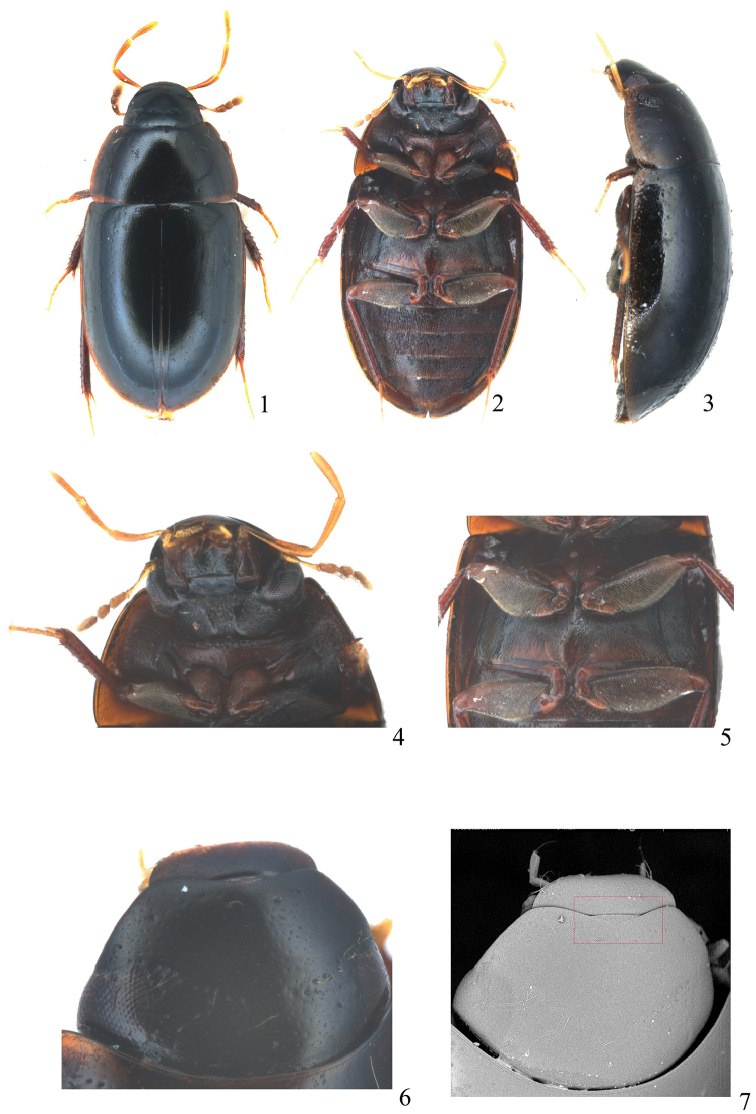
*Chasmogenus parorbus* sp. n. **1** dorsal habitus **2** ventral habitus **3** lateral habitus **4** head and prosternite, ventral **5** meso-, and metaventrite, ventral **6–7** Head, dorsal.

**Figures 8–10.**
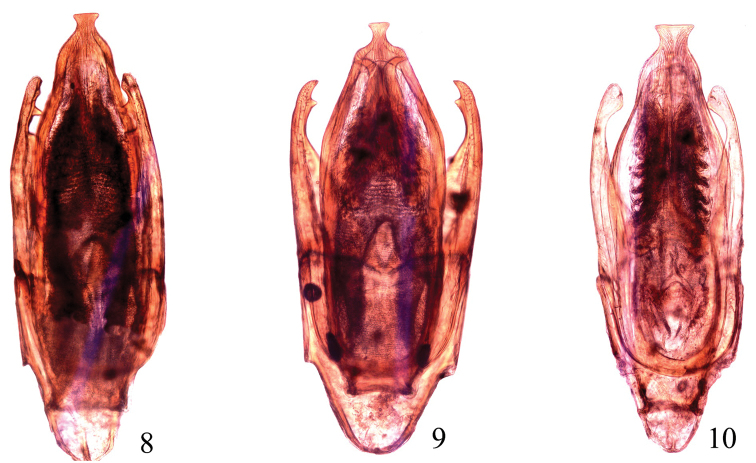
Aedeagi (dorsal view): **8** *Chasmogenus parorbus* sp. n. **9** *C. orbus* Wlatanabe **10** *C. abnormalis* Sharp.

Body oblong (Fig. 1). Black, pronotum and elytra with paler margins. Head, pronotum and elytra with distinct systematic punctures. Ground punctures of pronotum and elytra finer laterally and posteriorly than on disc (Figs 1, 3). Mesoventrite with a longitudinal and moderate high ridge posteromedialy (Fig. 5). Hind femora pubescent on basal four-fifths. Parameres shorter than median lobe, inner margin with a subapical tooth, apex obtuse rounded. Median lobe broad, gradually narrowing from apical fourth to apex, moderately curved on apical fifth and strongly curved subapically, apex truncate (Fig. 8).

#### Description.

Length 3.4–3.7 mm, width 1.7–2.0 mm. Body oblong, moderately convex. Dorsum of head, pronotum and elytra black with paler margins. Maxillary and labial palpi, and antennae uniformly yellowish brown (Figs 1–4). Ventral surface and legs reddish brown or dark brown.

***Head.*** Labrum sparsely and finely punctate, with slightly emarginate anterior margin and round arcuation on posterior margin. Clypeus emarginate anteriorly (Figs 6–7), systematic punctures distinct, ground punctures fine and sparse, with distance between punctures 1.5–3.0 × the width of one puncture. Frons with distinct systematic punctures, ground punctures as fine as those on clypeus but a little denser. Mentum transverse, ca. 2 × as wide as long, rugose, with distinct oblique sculptures and a few strong punctures, strongly depressed anteriorly, anterocentral notch developed (Fig. 4). Maxillary palpi obviously longer than width of head. Antenna with nine antennomeres, pedicel as long as antennomeres 3 to 6 combined.

***Thorax.*** Pronotum with distinct systematic punctures, ground punctures on disc as fine as those on frons, with distance between punctures 1.5–3.0 × the width of one puncture; lateral portions with finer punctures. Prosternite slightly bulged in middle, not carinate, with sparse pubescence, with transverse groove anteriorly (Fig. 4). Elytra with fine punctures similar to those of pronotum, systematic punctures distinct; with distinct sutural stria on posterior three-quarters. Mesoventrite with a longitudinal carina posteromedially, completely fused with mesanepisterna. Metaventrite pubescent with an irregular glabrous area (Figs 2, 5). Meso- and metafemoral pubescence on basal four-fifths.

***Abdomen.*** Abdominal ventrites densely pubescent. Apical margin of fifth ventrite with shallow emargination (Fig. 2).

***Aedeagus.*** Parameres shorter than median lobe, inner margin with a tooth subapically, apex obtuse rounded. Median lobe broad, gradually narrowing from apical quarter to apex, moderately depressed on apical fifth and strongly curved subapically, apex truncate (Fig. 8).

#### Remarks.

This species is close to *C. orbus* Watanabe, 1987. It can be distinguished from *C. orbus* by parameres obtuse apically, subapical tooth sharper; median lobe slender, slightly but distinctly constricted in apical quarter (Fig. 8).

#### Etymology.

The species name is combined from Latin “para-“, similar, and *orbus*, a species name of the genus.

#### Biology.

Aquatic, living in stagnant pools.

#### Distribution.

Only known from type locality.

### Chasmogenus orbus

Taxon classificationAnimaliaColeopteraHydrophilidae

Watanabe, 1987

Fig. 9

Helochares (Crephelochares) orbus Watanabe, 1987: 12 (for detailed description).

#### Material examined.

**CHINA: Hong Kong**: 2 males, 5 females (SYSU), Hong Kong, Rongshu’ao, 22°25.641'N, 114°17.410'E, 10m. 11.vi.2014, Fenglong Jia, Weicai Xie & Jiahuang Chen leg. [transcribed from Chinese].

#### Remarks.

Based on the original description of *C. orbus* by Watanabe (1987), the specimens here appear completely identical to this species. The senior author has dissected two males and sent photos of the aedeagus to Dr. Minoshima for comparison with Japanese specimens; he informed the senior author (via email) that he did not see any difference between the photos and the specimens from Japan. Similar to *C. parorbus* sp. n., it can be distinguished from this species by parameres being sharp apically, with the subapical tooth less sharp; and the median lobe broader, not constricted in apical quarter (Fig. 9).

#### Distribution.

China (Hong Kong); Japan. New for China.

### Chasmogenus abnormalis

Taxon classificationAnimaliaColeopteraHydrophilidae

(Sharp, 1890)

Fig. 10

Philydrus abnormalis Sharp, 1890: 351; Hansen 1999: 174 (complete synonymy); Devi et al. 2016: 296.

#### Material examined.

**CHINA: Guangdong**: 4 males, 6 females (SYSU), Guangdong, Zhuhai, Hengqing island, 10.VII.2006, Fenglong Jia leg. [transcribed from Chinese]. Macau: 2 females (SYSU), Cotai Ecosystematic Reserve, part 1, 8.iv.2014, Weicai Xie et Jinwei Li leg. [transcribed from Chinese].

#### Remarks.

This species is close to *C. orbus* from which it can only be distinguished by the aedeagus. Parameres without or with small subapical tooth (Devi et al. 2016: figs 10–13), apex broadened inwards (Fig. 10). Median lobe gradually narrowing from apical quarter to apex, moderately constricted in apical fifth and strongly curved subapically, apex truncate (Fig. 10).

#### Distribution.

China (Guangdong, Macau); Cambodia, India, Indonesia, Japan, Sri Lanka, Thailand, Vietnam.

##### Key to the species of the *Chasmogenus* of China

**Table:** 

1	Parameres with a distinct sharp subapical tooth on inner margin (Figs 8–9), not broadened inwards apically, median lobe narrowly truncate apically	**2**
–	Parameres with a small subapical tooth on inner margin (Fig. 10), broadened inwards apically, median lobe widely truncate apically	***C. abnormalis* (Sharp)**
2	Parameres with apex sharp and subapical tooth less sharp. Median lobe broader, not constricted in apical quarter (Fig. 9)	***C. orbus* Watanabe**
–	Parameres with apex obtuse and subapical tooth sharper. Median lobe relatively slender, distinctly constricted in apical quarter (Fig. 8)	***C. parorbus* sp. n.**

## Supplementary Material

XML Treatment for Chasmogenus

XML Treatment for Chasmogenus parorbus

XML Treatment for Chasmogenus orbus

XML Treatment for Chasmogenus abnormalis

## References

[B1] Clarkson B Ferreira NJR (2014) Four new species and first nominal record of *Chasmogenus* Sharp, 1882 (Coleoptera: Hydrophilidae) from Brazil. Zootaxa 3765(5): 481–494. https://doi.org/10.11646/zootaxa.3765.5.6 24870916 10.11646/zootaxa.3765.5.6

[B2] Devi MB Devi OS Fikáček M Minoshima Y Wanghengbam L (2016) Redescription and lectotype designation of *Chasmogenus abnormalis* (Sharp), with notes on its distribution. Zootaxa 4144(2): 296–300. https://doi.org/10.11646/zootaxa.4144.2.12 27470857 10.11646/zootaxa.4144.2.12

[B3] Fernández LA (1986) Consideraciones sobre el género *Chasmogenus* Sharp y descripción de *Chasmogenus sapucay* sp. nov. (Coleoptera: Hydrophilidae). Neotrópica 32: 189–193.

[B4] García M (2000) Cuatro nuevas especies de *Chasmogenus* Sharp, 1882 (Coleoptera: Hydrophilidae: Hydrophilinae) de Venezuela. Boletín del Centro de Investigaciones Biológicas Universidad del Zulia 34: 45–58.

[B5] Hansen M (1991) The hydrophiloid beetles: phylogeny, classification and a revision of the genera (Coleoptera, Hydrophiloidea). Biologiske Skrifter, Det Kongelige Danske Videnskabernes Selskab 40: 1–367.

[B6] Hansen M (1999) Hydrophiloidea (s. str.) (Coleoptera). World Catalogue of Insects 2. Apollo Books, Amsterdam, 416 pp.

[B7] Hebauer F (1992) The species of the genus *Chasmogenus* Sharp, 1882 (Coleoptera: Hydrophilidae). Acta Coleopterologica 8(2): 61–92.

[B8] Hebauer F (1995) Neues zu den Acidocerina Hansen (Helocharae d’Orchymont) der indomalaiischen Region (Coleoptera, Hydrophilidae). Acta Coleopterologica 11(3): 3–14.

[B9] Hebauer F (2001) Beitrag zur Kenntnis der Hydrophilidae von Neuguinea. Ergebnisse der zoologischen Forschungsreisen von M. Balke und L. Hendrich nach West Neuguinea (Irian Jaya) in den Jahren 1990–1998 (Coleoptera: Hydrophilidae). Acta Coleopterologica 17(1): 3–72.

[B10] Hebauer F (2002) New Hydrophilidae of the Old World (Coleoptera: Hydrophilidae). Acta Coleopterologica 18(3): 3–24.

[B11] Hebauer F (2006) Results of the Benin Mission 2001 and the Zambia Mission 2002 of F. & L. Kantner (Coleoptera: Hydrophilidae). Acta Coleopterologica 22(2): 11–24.

[B12] Komarek A Beutel R (2007) Phylogenetic analysis of Anacaenini (Coleoptera: Hydrophilidae: Hydrophilinae) based on morphological characters of adults. Systematic Entomology 32: 205–226. https://doi.org/10.1111/j.1365-3113.2006.00359.x

[B13] Sharp D (1882–87) Insecta Coleoptera Vol. 1, part 2 (Haliplidae, Dytiscidae, Gyrinidae, Hydrophilidae, Heteroceridae, Parnidae, Georissidae, Cyathoceridae, Staphylinidae). In: Godman FD, Salvin O (Eds) Biologia Centrali-Americana (16). 1–144. [1882]

[B14] Short AEZ (2005) A review of the subtribe Acidocerina with special reference to Costa Rica (Coleoptera: Hydrophilidae). Koleopterologische Rundschau 75: 191–226.

[B15] Short AEZ (2010) Hydrophilidae: Review of the subtribe Acidocerina of the Southwest Pacific islands (Coleoptera). In: Balke J (Ed.) Water Beetles of New Caledonia, volume 1. Monographs on Coleoptera 3: 297–318.

[B16] Short AEZ Fikaček M (2011) World catalogue of the Hydrophiloidea (Coleoptera): additions and corrections II (2006–2010). Acta Entomologica Musei Nationalis Pragae 51(1): 83–122.

[B17] Short AEZ Fikáček M (2013) Molecular phylogeny, evolution and classification of the Hydrophilidae (Coleoptera). Systematic Entomology 38: 723–752. https://doi.org/10.1111/syen.12024

[B18] Short AEZ Hebauer F (2006) World Catalogue of Hydrophiloidea – additions and corrections, 1 (1999–2005) (Coleoptera). Koleopterologische Rundschau 76: 315–359.

[B19] Watanabe N (1987) The Japanese species of Helochares (Crephelochares) (Coleoptera: Hydrophilidae), with Description of a New Species from Honshu. Aquatic Insects 9: 11–15. https://doi.org/10.1080/01650428709361262

[B20] Watts CHS (1995) Revision of the Australasian genera *Agraphydrus* Regimbart, *Chasmogenus* Sharp and *Helochares* Mulsant (Coleoptera: Hydrophilidae). Records of the South Australian Museum 28: 113–130.

